# Expression of Concern: Hotair contributes to cell proliferation and metastasis of cervical cancer via targeting miR-23b/MAPK1 axis

**DOI:** 10.1042/BSR-20171563_EOC

**Published:** 2021-04-06

**Keywords:** Cervical cancer, HOTAIR, MAPK1, miR23b

The Editorial Office has been made aware of potential issues surrounding the scientific validity of this paper, hence has issued an expression of concern to notify readers whilst the Editorial Office investigates.

